# Kidney Disease in Disadvantaged Populations

**DOI:** 10.1155/2012/469265

**Published:** 2012-04-09

**Authors:** David Martins, Lawrence Agodoa, Keith Norris

**Affiliations:** ^1^Clinical and Translational Research Center, College of Medicine, Charles R. Drew University of Medicine and Science, 1731 East 120th Street, Los Angeles, California 90059, USA; ^2^Office of Minority Health Research Coordination, NIDDK, National Institutes of Health, Building 2DEM, Room 902, 6707 Democracy Blvd., Bethesda, Maryland 20892, USA; ^3^Division of Nephrology, Department of Medicine, Charles R. Drew University of Medicine and Science, Clinical Research Center Annex, 11705 Deputy Yamamoto Place, Los Angeles, California 90262, USA

## Abstract

Disadvantaged populations across the globe exhibit a disproportionate burden of chronic kidney disease (CKD) because of differences in CKD occurrence and outcomes. Although many CKD risk factors can be managed and modified to optimize clinical outcomes, the prevailing socioeconomic and cultural factors in disadvantaged populations, more often than not, militate against optimum clinical outcomes. In addition, disadvantaged populations exhibit a broader spectrum of CKD risk factors and may be genetically predisposed to an earlier onset and a more rapid progression of chronic kidney disease. A basic understanding of the vulnerabilities of the disadvantaged populations will facilitate the adaptation and adoption of the kidney disease treatment and prevention guidelines for these vulnerable populations. The purpose of this paper is to examine recent discoveries and data on CKD occurrence and outcomes in disadvantaged populations and explore strategies for the prevention and treatment of CKD in these populations based on the established guidelines.

## 1. Background and Epidemiology

The global prevalence of chronic kidney disease (CKD) is increasing and creating enormous socioeconomic burdens for patients, families, society, and the health care system across the globe. Data from the third National Health and Nutrition Examination Survey (NHANES 1999–2004) suggest that about 1 out of 8 adult Americans exhibit evidence of CKD [1]. Comparable estimates have been reported in Asia [2], Australia [3], and across Europe [4–6]. The lack of national registries and limited representative national surveys in developing countries make the estimation of the burden of CKD in these countries difficult. However, the risk factors for CKD are known to be just as prevalent in many developing countries as in the developed countries. Therefore, the burden of CKD in those developing countries may be comparable to those of the developed countries. In addition, developing countries exhibit a disproportionate burden of infectious and environmental factors that broaden the spectrum of CKD risk factors and is apt to increase CKD burden. A greater understanding of CKD onset and progression among racial/ethnic minorities and socioeconomically disadvantaged persons in the US may provide insights into CKD burdens in similar populations globally.

 The Kidney Disease Outcomes Quality Initiative (KDOQI) guidelines by the National Kidney Foundation in 2002 defined CKD as functional and structural abnormalities of the kidneys that persist for more than three months. This widely publicized and generally accepted guidelines included the presence of markers of kidney damage such as albuminuria in the definition of CKD and established five progressive stages based on a sustained reduction in the estimated glomerular filtration rate (eGFR) with specific evaluation and treatment recommendations [7]. (Table 1) This expanded definition of CKD allows for the identification of CKD in its earliest stages when the eGFR might still be well within the normal limits and is critical to early detection and treatment of CKD.

 There is a dearth of population-based prevalence data on the different stages of CKD across the globe. In the United States (US), the National Health and Examination Survey estimated that the prevalence of CKD stages from 1 to 4 increased from 10.0% (95% confidence interval [CI], 9.2%–10.9%) in 1988–1994 to 13.1% (95% CI, 12.0%–14.1%) in 1999–2004 with a prevalence ratio of 1.3 (95% CI, 1.2–1.4). The specific prevalence estimates for CKD stages 1 to 4 in 1988–1994 and 1999–2004 are as shown in Table 1 [1]. Although the CKD prevalence data across Europe are comparable to those of the US, the progression of CKD to treated end-stage renal disease (ESRD) is generally slower in Europe than in the USA [8]. 

## 2. Risk Factors and Rate of Progression

The increasing prevalence of diabetes across the nations is the greatest risk factor for CKD in the world. It has been estimated that there would be 366 million adults with diabetes worldwide by the year 2030 [9]. The prevalence of diabetes in developing countries is rapidly approaching that of developed countries. In Mexico, the prevalence of diabetes is as high as 25% among 25 to 40-year-old Mexicans [10]. The rising rates of diabetes in developing countries will engender a disproportionate burden of CKD in these disadvantaged populations. Diabetic nephropathy is becoming increasingly recognized as the leading cause of CKD in both the developed and many developing countries. In fact, diabetic nephropathy accounts for 65% of the ESRD in Puerto Rico [11] and is a common cause of ESRD in many countries in Africa and the Middle East [12].

 The onset and progression of CKD vary from one etiology to another and from patient to patient, even with the same etiology. Regardless of the etiology, established CKD can accelerate its own course by inducing cardiovascular (CV) disease and metabolic complications. The risk of this CV disease and hence the rate of CKD progression is generally higher at stages 3–5 than at earlier stages of CKD [13]. The presence of multiple risk factors such as hypertension and lipid disorders is also apt to promote an earlier onset and a more rapid progression of CKD, and may explain the fact that hypertension and diabetes account for two-thirds of the ESRD in the United States [14]. Disadvantaged populations, particularly in developing countries, frequently exhibit multiple risk factors for CKD and harbor nontraditional risk factors such as schistosomiasis, tuberculosis and amyloidosis [15]. Environmental pollution, pesticides, analgesic abuse, herbal medications, and unregulated food additives also contribute to the disproportionate burden of CKD in many disadvantaged populations worldwide [16].

**Figure 1:**
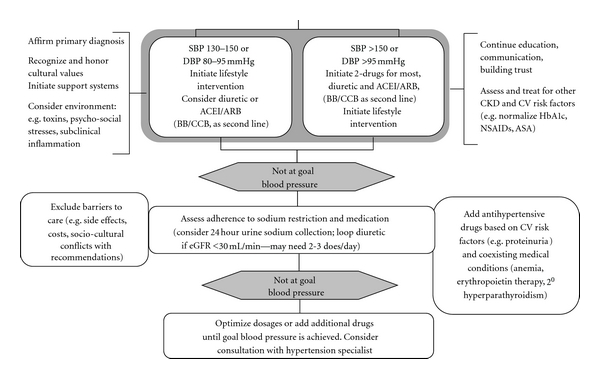
Algorithm for a comprehensive approach to hypertension control in disadvantaged persons with chronic kidney disease (CKD). SBP; systolic blood pressure; DBP; diastolic blood pressure; BB; beta blocker; ACEI; angiotensin converting enzyme inhibitor; ARB; angiotensin receptor blocker; CV; cardiovascular; CCB; calcium channel blocker; eGFR; estimated glomerular filtration rate. Adapted from Martins et al. [27].

 The progression of CKD to ESRD has been reported to be more rapid in the USA than in Europe. Within the USA however, the prevalence of early CKD is comparable across racial/ethnic categories but the progression of CKD to ESRD is far more rapid among minority populations, with ESRD rates nearly 4-fold higher among African Americans in comparison to US Whites, despite similar prevalence rates of early CKD [17]. The rapid progression of CKD to ESRD among minority populations in the USA is largely attributable to higher prevalence and greater severity of diabetes and hypertension, lower socioeconomic status, lesser access to care, excess exposure to environmental toxins, and other factors [18]. Compared with Whites, African Americans have much higher rates and earlier onset of diabetes and hypertension and exhibit greater rates of diabetic and hypertensive complications such as CKD, stroke, and heart disease [19]. In spite of the effectiveness of the control of serum glucose and blood pressure levels to mitigate the progression of diabetic nephropathy [20], the overall blood pressure control remains unacceptably low ranging from 50% in the USA [21] to about 64% in Canada [22]. The pathologic synergy of hypertension with diabetes as well as the higher rate of hypertension and the lower rate of blood pressure control may contribute to the more rapid progression of CKD to ESRD amongst African Americans. Given the high prevalence of hypertension, particular attention to its control is paramount for preventing CKD initiation and progression (Figure 1).

 Although the pathophysiologic basis for the variation in the progression of CKD to ESRD across populations is probably multifactorial and currently poorly understood, it is becoming increasingly apparent that gene-based differences in disease profile [23] may contribute to the disproportionate burden of CKD across populations. A few rare kidney diseases exhibit monogenic abnormalities with Mendelian patterns of inheritance but genetic variations are becoming increasingly associated with an increased risk of developing common kidney diseases in population-based genetic studies. Genome-wide admixture mapping studies have recently revealed variations in the regions of MYH9 and APOL 1 on chromosome 22 that protect against a lethal form of African sleeping sickness but are highly associated with an increased risk of nondiabetic CKD [24] and may explain as much as 70% of the differences in the rates of ESRD between US Whites and African Americans [25]. In addition, genetic differences have been known to modulate ethnic responses to therapeutic agents and may contribute to differences in CKD outcomes across racial and ethnic lines [26]. The understanding of the epidemiologic, genetic, and socio-cultural nuances of CKD among disadvantaged populations worldwide will facilitate the development of appropriate treatment strategies that will optimize the clinical outcomes in these vulnerable populations. 

## 3. Evaluation and Treatment

The earliest stage of CKD is characterized by the presence of microalbuminuria and a normal eGFR. This subtle manifestation of CKD has been associated with a 25- to 40-fold increase in the risk for ESRD and carries comparable risks of developing CV disease and ESRD as stage 3 CKD [28]. In an analysis of persons with optimal and high-normal BP, there was no significant difference in the risk of microalbuminuria among Whites, but a trend toward increased risk of microalbuminuria among Mexican Americans (OR 1.16; CI 0.90–1.51), and a significantly increased risk of microalbuminuria among African Americans (OR 1.30; CI 1.04–1.64) was observed [29]. The greater risk of proteinuria exhibited by African Americans at any given level of increased BP may contribute in part to the nearly fivefold greater increase in the overall incidence of hypertension-related ESRD among African Americans compared to Whites, as well as the more than 15 times greater rates of hypertension-related ESRD for young African-American men between the ages of 20 and 44 compared to their White counterparts [30].

Cardiovascular risk factors (Table 2) and the presence of CV disease accelerate the progression of CKD and confer additional risk of mortality [31]. Conversely, all stages of CKD are associated with an increased risk for CV death and complications [32]. A substantial portion of the etiologic suppositions and therapeutic strategy in disadvantaged populations revolve around the important role of the rennin-angiotensin system (RAS) in the modulation of hypertension and the mediation of the hypertension-related complications. The documented role of RAS as a facilitator of the progression of CKD engendered the expectation of an attenuated risk of hypertension-related end-organ damage in populations with low-renin hypertension. But contrary to this expectation, many African Americans with high rates of sodium sensitivity and low plasma renin levels experience more severe hypertension-related end-organ complications such as proteinuria and cardiorenal disease [33]. The dissociation of the circulating RAS from the intrarenal RAS has been suggested as a probable mechanism for this unusual experience based on the observation that upregulation of the intrarenal RAS accompany renal interstitial inflammation and oxidative stress in the kidneys and cardiovascular tissues of salt-sensitive rats fed a high-salt diet [34]. Despite the low circulating renin level, RAS blockade reversed endothelial dysfunction,attenuated proteinuria, and reduced renal injury independent of blood pressure changes in animal models [35], making RAS inhibition a rational therapeutic strategic option for low renin hypertension in CKD, particularly in African Americans with CKD where local RAS upregulation in the kidney could exacerbate both diabetic and hypertensive CKD [27].

The effectiveness of this therapeutic strategy has been demonstrated in the large prospective African American Study of Kidney Disease and Hypertension (AASK) that examined the effects of two levels of blood-pressure control (standard: ~135–140/85–90 mmHg and intensive: ≤120/80 mmHg) and three classes of initial antihypertensive therapy (Angiotensin Converting Enzyme [ACE] inhibitor, beta blocker or calcium channel blocker) on the progression and outcomes of hypertensive renal disease, excluding individuals with substantial proteinuria (>2.5 g per day), diabetes, or other causes of CKD and established that the development of ESRD, doubling of serum creatinine, or death was less frequent in the ACE inhibitor group than in the beta-blocker or calcium-channel-blocker groups [36]. Although there was no difference in the progression of CKD between the blood pressure level groups in the original study, a subsequent follow-up study demonstrated a potential benefit of blood pressure less than 130/80 mmHg among the participants with protein to creatinine excretion ratio greater than 0.22 (hazard ratio, 0.73; *P* = 0.01) at baseline [37]. 

## 4. Secondary Prevention

The prevention of CKD has to be part of a comprehensive CV disease prevention strategy to be affordable and cost-effective particularly among disadvantaged populations. Many of the risk factors for CV disease are behavioral and modifiable (Table 3). The identification and communication of the risk attributable to health beliefs and behaviors within the context of overall CV disease burden and risk for CKD should engage and encourage the patient to be proactive in risk reduction strategies. The inclusion of additional culturally appropriate healthcare professionals (e.g., a dietitian, pharmacist, and social worker) and/or family members can be an effective strategy to facilitate communication and reinforce recommended therapeutic lifestyle changes. The KDOQI Clinical Practice Guidelines on Hypertension and Antihypertensive Agents in Chronic Kidney Disease recommend initial antihypertensive therapy with an ACE inhibitor or an Angiotensin Receptor Blocker (ARB) for patients with CKD, regardless of ethnicity recognizing that many will require combination therapy with a diuretic [38]. The evaluation of response to therapy should include not only checking that blood pressure is less than the recommended target of 130/80 mmHg but assessing complications and monitoring the change in the level of proteinuria, which is a powerful predictor of progression of hypertensive kidney disease in all patients at any given eGFR in all patients [39]. While the cost effectiveness of screening the general population with microalbuminuria is debatable, it is generally accepted as reasonable to target individuals with cardiovascular risk factors for CKD screening using microalbuminuria.

## 5. Conclusion

The management of CKD in disadvantaged populations requires a comprehensive approach and a detailed attention to the prevailing socioeconomic and cultural factors that often militate against optimum clinical outcomes in these vulnerable persons. Lessons learned from racial/ethnic minorities and socioeconomically disadvantaged persons in the USA may provide insights into the care of similar populations globally. It is our recommendation that the initial evaluation of patients with CKD be broad enough to uncover nontraditional risk factors for CKD and include a comprehensive cardiovascular assessment. We reiterate that the initial therapy for treating hypertension and/or proteinuria in all patients with CKD comprise RAS inhibition with diuretic, because this combination appears most effective to achieve BP control and to confer additional cardiorenal protection beyond that offered by blood-pressure control alone. However, the overall treatment decision should be guided by individual response, coexisting risk factors and potential cultural/socioeconomic considerations such as cost of medications and insurance coverage, which affect adherence to both pharmacologic and nonpharmacologic interventions.

##  Disclosures

K. Norris has declared associations with the following companies: Abbott, Amgen, Merck, Monarch Pharmaceuticals, and Pfizer. The other authors declared no conflict interests.

## Figures and Tables

**Table 1:** Stages of chronic kidney disease.

Stage	Description	eGFR (mL/min/1.73m^2^)	Prevalence estimates,1988–1994	Prevalence estimates,1999–2004
1	Slight kidney damage with normal or increased filtration	More than 90	1.7% (95% CI 1.3%–2.2%)	1.8% (95% CI 1.4%–2.3%)
2	Mild decrease in kidney function	60–89	2.7% (95% CI 2.2%–3.2%)	3.2% (95% CI 2.6%–3.9%)
3	Moderate decrease in kidney function	30–59	5.4% (95% CI 4.9%–6.0%)	7.7% (95% CI 7.0%–8.4%)
4	Severe decrease in kidney function	15–29	0.21% (95% CI 0.15%–0.27%)	0.35% (95% CI 0.25%–0.45%)

Data from [1].

**Table 2:** Cardiovascular disease risk factors associated with CKD progression.

Modifiable

High blood pressure
Dyslipidemia (e.g., elevated LDL, decreased HDL)
Diabetes mellitus
Smoking
Overweight and obesity
Atherosclerosis
Coronary artery disease
Congestive heart failure

Unmodifiable

Age (≥65 years)
Family history of premature CVD
Male gender
Menopause
US racial ethnic minority status (African Americans, American Indians, and Asian Americans)

Data from [7].

**Table 3:** Life style modifications for cardiovascular risk reduction.

Goals	Lifestyle Modifications
Weight loss	Lose weight gradually by making permanent changes in daily diet for the entire family. Initiate a 10 kcal per pound of body weight per day diet. Set a reasonable weight loss goal (1-2 lb/week for first 3–6 months).
Dietary goals: Low fat Low sodium High potassium High calcium	Eat more broiled and steamed foods. Eat more grains, fresh fruits, and vegetables. Eat fewer fats and use healthier fats, such as olive oil. Eat fewer processed foods, fast foods, and fried foods. Read labels and pay attention to the sodium salt and fat content of foods. Do not season foods with smoked meats, such as bacon and ham hocks. If lactose intolerant, try lactose-free milk or yogurt, or drink calcium-fortified juices, or soy milk.
Physical fitness	Increase physical activity as part of the daily routine: e.g., if currently sedentary, get off the bus 6 blocks from home or walk in the evening with spouse, friend or group. Gradually increase time spent at an enjoyable physical activity to 30–45 minutes 3–5 days/week.
Stress management	Learn stress reduction techniques and coping skills for specific stressors in the work and/or home environment. Meditation, Relaxation, Yoga, Biofeedback, others.
Smoking cessation	Stop smoking and advocate for a smoke-free environment
Alcohol moderation	Drink no more than 2 beers, 1 glass of wine, or 1 shot of hard liquor per day (50% less for women).

Adapted from Martins DS and Norris KC. Hypertension treatment in African-American: Physiology is less important than sociology. Cleveland Clinic Journal of Medicine. 2004; 71(9) 735-743.
